# The war on cryptococcosis: A Review of the antifungal arsenal

**DOI:** 10.1590/0074-02760170391

**Published:** 2018-02-19

**Authors:** Ahmad Mourad, John R Perfect

**Affiliations:** Duke University Medical Center, Department of Medicine, Division of Infectious Diseases, Durham, NC, USA

**Keywords:** cryptococcosis, Cryptococcus, cryptococcal meningitis, antifungal drugs

## Abstract

Cryptococcal meningitis is the most common central nervous system infection in the world today. It occurs primarily, but not exclusively, in immunocompromised individuals and despite substantial improvement in management of clinical events like AIDS, the numbers of cases of cryptococcosis remain very high. Unfortunately, despite several antifungal agents available for treatment, morbidity and mortality rates remain high with this fungal infection. In this Review, we will describe the treatments and strategies for success, identify the failures, and provide insights into the future developments / improvements for management. This sugar-coated yeast can play havoc within the human brain. Our goals must be to either prevent or diagnose disease early and treat aggressively with all our clinical tools when disease is detected.

Cryptococcosis is a global invasive mycosis that is associated with high morbidity and mortality. Patients with HIV infection are at a significantly increased risk of developing this fungal disease. With its profound propensity to locate within the central nervous system (CNS), *Cryptococcus spp.* frequently causes fungal meningitis. In fact, this encapsulated yeast remains the most common cause of meningitis in HIV-infected individuals living in sub-Saharan Africa. It is estimated that in 2014 there were over 220,000 new cases of cryptococcal meningitis globally resulting in more than 180,000 deaths and is responsible for 15% of all AIDS-related deaths. Although the annual rate of cryptococcal disease has decreased after the widespread use of highly active anti-retroviral therapy (HAART) in developed countries, the prevalence of cryptococcal infection remains at a high level in low and middle-income countries despite the availability of HAART (Tenforde et al. 2017). The one-year mortality after cryptococcal meningitis ranges from 10-30% in North America to up to 50-100% in low-income countries (Rajasingham et al. 2017, Williamson 2017, Williamson et al. 2017).

Furthermore, non-HIV patient populations are also at risk of cryptococcal infection, notably transplant recipients and patients on immunosuppressive therapies. For example, approximately 2-3% of solid organ recipients have been found to develop cryptococcal infection with most patients presenting with disseminated infection (Larsen et al. 1994, Mayanja-Kizza et al. 1998, Milefchik et al. 2008, Pappas et al. 2009). With over 33,000 solid organ transplants performed in the United States alone in 2016, the number of cryptococcal infection cases remains unacceptably high (HRSA 2017). With our increasing use of immune-modulators from corticosteroids, biological modifiers (i.e. anti-TNF and anti-CD54) to new anti-cancer agents such as ibrutinib (Messina et al. 2017), we can expect the number of patients with cryptococcosis to remain concerning (George et al. 2017).

Antifungal drug therapy remains the mainstay of treatment of these cryptococcal infections. This review aims at highlighting the drugs and strategies utilized for the management of this life- threatening infection, as well as the new developments in treatment.

*Therapeutic principles for cryptococcal meningitis* - Before examining the details of our arsenal, several therapeutic principles for management of cryptococcal meningitis should be listed: (1) early diagnosis is helpful to a successful outcome of treatment with a lower burden of yeasts and less destruction from a persistent, dysregulated immune system; (2) identification of new, old, and changing risk factors is necessary to properly utilize our outstanding biomarkers for disease; (3) a fungicidal anticryptococcal regimen that rapidly clears viable yeasts from the subarachnoid space is optimal management; (4) major complications of cryptococcal meningitis should be carefully identified and managed. These include (a) increased intracranial pressure and (b) development of the immune reconstitution inflammatory syndrome (IRIS); (5) further understanding of *in vitro* anticryptococcal yeast susceptibility testing for resistance (there are no validated drug break points) and strain evaluation genetically for identification of possible “bad actor” strains will be helpful. This area requires further research to become more clinically relevant and precise; (6) we must control the concomitant underlying diseases at all costs and this will likely demand attention to the “Goldilocks’s Paradigm of Immunology, not too much and not too little but must get it just right”; (7) our goal is to reduce mortality but it must also be accompanied by a reduction in morbidity, which is less chronicled in present reviews.

*Amphotericin B & flucytosine (5-FC)* - The polyene, amphotericin B, has been the mainstay of treatment of cryptococcal meningitis in HIV-infected individuals and transplant recipients as well as non-HIV and non-transplant patients for several decades. Using a polyene-based regimen has been associated with significant reduction of the yeast burden within the CNS and is correlated with improved survival (Sloan & Parris 2014). Furthermore, the combination of amphotericin B with 5-FC has been shown to be the most fungicidal regimen at present in the clinics and is associated with improved survival among those with cryptococcal meningitis compared to amphotericin B treatment alone (Larsen et al. 1990 d, de Gans et al. 1992, Brouwer et al. 2004, Dromer et al. 2008, Day et al. 2013, Sloan & Parris 2014). Recently, a large randomized study (ACTA) with over 700 patients confirmed the superiority of this combination (Kanyama & Molloy 2017). In medically-developed countries, using the combination of amphotericin B and 5-FC for induction of HIV-infected patients with cryptococcal meningitis in a three-part strategy of induction-consolidation-maintenance is recommended at a dose of amphotericin B deoxycholate of 0.7-1 mg/kg/day intravenously with 5-FC 100 mg/kg/day orally for at least two weeks (Perfect et al. 2010). Studies that have assessed the use of the higher dose of amphotericin B (1 mg/kg/day vs. 0.7 mg/kg/day) with 5-FC found that despite the improved fungicidal activity, the number of serious adverse events related to higher doses increased (Bicanic et al. 2008).

Substituting lipid formulations of amphotericin B for amphotericin B deoxycholate is favored in most patients in medically developed countries and particularly in transplant recipients who are at significantly higher risk of nephrotoxicity due to their potential concomitant use of other nephrotoxic drugs such as calcineurin inhibitors (Coker et al. 1993, Sharkey et al. 1996, Leenders et al. 1997, Baddour et al. 2005, Singh et al. 2005, Hamill et al. 2010). Liposomal amphotericin B or amphotericin B lipid complex are recommended at a dose of 3-4 mg/kg/day or 5 mg/kg/day, respectively (Perfect et al. 2010). Extending the induction period depends on the prognosis and response of the individual at the time. In general, there is a switch toward primary use of lipid products of amphotericin B in resource-available health care systems and shorter course amphotericin B therapy (one week) in resource-limited healthcare systems for the induction period.

*Azoles: fluconazole* - Fluconazole is used in the three-part strategy of induction-consolidation-maintenance in combination with amphotericin B as a substitute for 5-FC in the induction phase when 5-FC is not available as well as alone in the consolidation and maintenance phases for cryptococcal meningitis (Perfect et al. 2010). Fluconazole has a fungistatic effect on cryptococcal meningitis and so the burden of yeast must be low or dose of fluconazole must be very high for it to have a significant therapeutic impact in the CNS (Martinez et al. 2000, Rollot et al. 2001, Aberg et al. 2002, Vibhagool et al. 2003, Mussini et al. 2004). For this reason, reduction of fungal burden with amphotericin B in combination with fluconazole is necessary for successful outcome in resource-limited settings (Saag et al. 1992, Haubrich et al. 1994, Menichetti et al. 1996, Robinson et al. 1999, Bicanic et al. 2007). Although amphotericin B and fluconazole in combination are not as potent as amphotericin B plus 5-FC, it clearly can effectively be used in induction therapy at higher doses of fluconazole of ≥ 800 mg/day (Pappas et al. 2009). The use of high dose fluconazole as monotherapy for induction therapy is still associated with significantly higher mortality than combination therapy and needs further investigation to implement successful monotherapy regimens in resource-limited settings (Nussbaum et al. 2010, Gaskell et al. 2014). However, the use of fluconazole with 5-FC in combination as an all oral regimen continues to be studied and most recent data suggest that it is not significantly inferior to the amphotericin B- containing regimens (Kanyama & Molloy 2017).

Following induction therapy, relatively high doses of fluconazole are used in the consolidation phase for a recommended period of eight weeks and this has been shown to be superior to other azoles such as itraconazole (Perfect et al. 2010, Bicanic et al. 2015).

The last phase of the three-part strategy, suppression or maintenance, is also continued with fluconazole if the patient is stable after consolidation. The use of a suppression strategy has been validated by several studies, due to the high relapse rate of cryptococcal meningitis prior to HAART (15%) (Perfect 2016). Fluconazole was shown to be superior to itraconazole and even weekly amphotericin B for suppression (Perfect 2016). Suppression with fluconazole is generally continued for at least one year in HIV patients and for 6-12 months in non-HIV patients (Perfect et al. 2010). In HIV patients, after successful introduction of HAART, discontinuing suppressive therapy with ﬂuconazole is recommended when CD4 ≥ 100 cell/µL and an undetectable HIV RNA level is sustained for ≥ 3 months (Perfect et al. 2010).

For other cryptococcal infections, such as pulmonary cryptococcosis, fluconazole monotherapy is recommended as treatment for mild to moderate disease (Perfect et al. 2010).

*Itraconazole, voriconazole and posaconazole* - The extended-spectrum azoles have a limited experience in treatment of cryptococcosis. Itraconazole has successfully treated cryptococcal meningitis but its poor absorption and limited CNS penetration makes it unreliable (Chotmongkol & Jitpimolmard 1992, de Gans et al. 1992, Pitisuttithum et al. 2005). Voriconazole, which does penetrate into the CNS well, has had some success in primary treatment, particularly in normal hosts (Yao et al. 2015). Posaconazole has excellent activity against *Cryptococcus spp.* but has limited CNS penetration. The largest series of cryptococcal meningitis treatment with posaconazole had 14/29 (48%) success (Pitisuttithum et al. 2005).

*Isavuconazole* - The VITAL study was an open label phase III trial to assess the use of oral and intravenous isavuconazole for primary or salvage therapy of *Cryptococcus spp.* and dimorphic mycoses infections. Of the nine patients with cryptococcal infections receiving isavuconazole, 67% had either success, partial success or stable disease with an all-cause mortality of 11%. Furthermore, preliminary data suggests that isavuconazole has adequate CNS penetration and might likely have a role in treating cryptococcal meningitis and CNS infections, but further studies are necessary. Adverse events occurred at around 37% with this azole but were more favorable than amphotericin B and none of the patients discontinued therapy due to adverse events (Thompson 3rd et al. 2016).

*HAART* - HAART during management of cryptococcal meningitis is a double-edged sword. On the one hand, it could improve the immune response to the infection but on the other, it might contribute to IRIS as well as possess drug interactions and toxicity to curtail the management plan. Deciding when to initiate HAART is critical. Delaying HAART for five weeks after diagnosis and initiation of antifungal therapy improved survival compared to only one-two weeks (Zolopa et al. 2009) and clearly, early use of HAART in first two weeks of cryptococcal meningitis treatment increased deaths (Boulware et al. 2014). Current recommendations suggest delaying HAART for two-10 weeks after initiation of antifungal therapy (Perfect et al. 2010). Furthermore, in resource-limited countries with difficulty implementing wide spread HAART for HIV patients, initiating HAART within four-five weeks might be required to adequately control the severe underlying disease.

*Combination screening & pre-emptive therapy* - Cryptococcal antigen (CrAg) can be detected in serum more than three weeks prior to onset of symptoms of cryptococcal meningitis. Global cryptococcal antigenemia in 2014 was estimated at 6% of HIV-infected patients with CD4 counts less than 100 cells/µL, translating to approximately 280,000 individuals (Rajasingham et al. 2017). Asymptomatic cryptococcal antigenemia is well known to occur in patients with HIV infection (Longley et al. 2016). Furthermore, HIV-infected patients with low CD4 counts who have a positive CrAg have significantly higher mortality (Desmet et al. 1989, Nelson et al. 1990, Tassie et al. 2003, Liechty et al. 2007, Micol et al. 2007). As a result, the World Health Organization (WHO) recommended that HIV patients with CD4 ≤ 100 cells/µL should be tested for cryptococcal antigenemia (McKenney et al. 2015). If tested positive, asymptomatic patients would be given oral fluconazole for pre-emptive antifungal therapy unless titer was high (≥ 1:160) in which case CSF should be examined for presence of meningitis (Letang et al. 2015, McKenney et al. 2015). This strategy was shown to not only decrease cryptococcal disease and improve survival, but has proven cost-effective in resource-limited countries with high incidences of disease (Meya et al. 2010, Kaplan et al. 2015, McKenney et al. 2015). The development of cheaper lateral flow assays can be utilized with an accuracy of nearly 100% to diagnose cryptococcal disease and even suggest prognosis, with each 2-fold increase in titers associated with higher mortality at two and 10 weeks (Kabanda et al. 2014). Therefore, in resource-limited areas with high antiretroviral drug resistance and significant prevalence of cryptococcal disease, a CrAg screening and preemptive therapy strategy should be considered (Ssekitoleko et al. 2013).

*Immunotherapy* - Despite the abundance of in-vitro and in-vivo studies demonstrating the significant contribution of immune modulation to the course of cryptococcal infections, there is a lack of sufficient clinical data to make strong recommendations for the use of immunotherapy in treating these infections (Antachopoulos & Walsh 2012). In murine models of cryptococcal infections, administering IL-12 and IL-18 have been shown to significantly reduce the fungal cell burden in many organs and enhance elimination of *Cryptococcus spp..* These murine models have also shown an increase in IFN-γ after administering IL-12 or IL-18, with anti IFN-γ antibodies eliminating the protective effect of the interleukins (Kawakami et al. 1996b, 1997). Direct administration of IFN-γ prolonged survival in murine models of cryptococcosis (Kawakami et al. 1996a). Administration of TNF-α in-vitro increased the anticryptococcal activity of macrophages and combining TNF-α with granulocyte-macrophage colony stimulating factor enhanced phagocytosis by murine macrophages (Collins & Bancroft 1992, Kawakami et al. 1999). Murine models using TNF-α related antibodies decreased fungal cell burden, mediated by an increase in IFN-γ (Zhou et al. 2006). A study of 62 HIV patients receiving antifungal therapy for cryptococcal meningitis showed that survivors compared to non-survivors had higher CSF concentrations of IFN-γ, TNF-α, IL-6 and 8 (Siddiqui et al. 2005). Furthermore, some non-HIV, non-transplant and supposedly immunocompetent patients with cryptococcal disease have been shown to possess autoantibodies to GM-CSF (Saijo et al. 2014).

With this very supportive background for immunotherapy, several human studies have been performed. First a phase II trial evaluating the use of adjuvant recombinant IFN (rIFN)-γlb in HIV patients with cryptococcal meningitis showed accelerated clearance of CSF cultures but results did not achieve statistical significance (Pappas et al. 2004). Second, another recombinant interferon-gamma study showed a positive effect on the reduction of yeasts in the CSF without adverse effects (Jarvis et al. 2012). Despite these two positive studies for adjunct recombinant gamma interferon current guidelines have a low level recommendation for the addition of rIFN-γ to the antifungal regimen of patients with persistent cryptococcal infection (Perfect et al. 2010). This hesitancy to elevate immunomodulation therapy as first line therapy in the induction regimen may be due to concerns about monitoring for appearance of IRIS in CNS infections and the general lack of robust clinical trials.

Other immunotherapeutic options being studied involve the use of monoclonal antibodies targeting virulence factors of *Cryptococcus spp.* such as capsule, as well as radiolabelled monoclonal antibodies to deliver radiation to cryptococcal cells and induce an apoptosis-like cell death (Larsen et al. 2005, Antachopoulos & Walsh 2012).

*Adjuvant steroid use* - Given that adjuvant steroid use in patients with other types of meningitis like tuberculosis and some types of bacterial meningitis has been shown to reduce morbidity and mortality, a large clinical trial was performed to evaluate the combination of dexamethasone with amphotericin B and fluconazole antifungal therapy. The trial was stopped early due to the increased mortality at 10 weeks and six months as well as more adverse events in the group receiving dexamethasone compared to placebo. The adverse events included progression of infections, nephrotoxicity and cardiac toxicity. Furthermore, the addition of corticosteroids reduced the fungicidal activity of the drugs (Beardsley et al. 2016). However, it is important to note that this study evaluated the routine administration of dexamethasone at the beginning of therapy and not its use in management of IRIS. There have been some expert opinions on the use of steroids to curb intrathecal inflammation after achieving microbiological control (Panackal et al. 2016). A study involving patients with cryptococcal spinal arachnoiditis showed that excessive inflammation prolonged symptoms that later improved with administration of corticosteroids (Panackal et al. 2017). Therefore, despite the disadvantages of routine use of dexamethasone in treatment of cryptococcal meningitis, it is important to recognize that steroid therapy may be life-saving during IRIS. A taper of corticosteroids can be considered over two-six weeks, based mainly on expert opinions.

*Lumbar puncture (LP)* - Studies have shown that raised intracranial pressure (ICP) in cryptococcal meningitis is associated with increased mortality (Graybill et al. 2000). The withdrawal of CSF appears to be effective in reducing intracranial pressure caused by an outflow obstruction of the arachnoid villi by clumping yeasts (Denning et al. 1991). A lumbar puncture to control ICP and symptoms has been recommended and a study evaluating the impact of therapeutic LPs on survival showed a 69% relative improvement associated with therapeutic LPs irrespective of initial ICP, suggesting that any patient with cryptococcal meningitis may benefit (Perfect et al. 2010, Rolfes et al. 2014). Hence, utilizing therapeutic LPs in the management of cryptococcal meningitis must be considered at times but its precise use and frequency are not yet defined.

*New strategies: advancing cryptococcal meningitis treatment for Africa (ACTA)* - ACTA is a phase III trial aimed at finding more feasibly implemented regimens for resource-limited healthcare systems and to determine if 5-FC or fluconazole is better adjuvant treatment. Three treatment strategies were compared: (1) an all oral strategy, of high-dose fluconazole and 5-FC for two weeks; (2) amphotericin B plus either high-dose oral fluconazole/5-FC for one week only; (3) amphotericin B plus high dose fluconazole / 5-FC for two weeks (control). An early presentation from the trial revealed that the short course arms of amphotericin B and the all-oral fluconazole/5-FC were non-inferior to the control group. Furthermore, using 5-FC as adjuvant therapy with amphotericin B led to lower mortality compared with fluconazole. The one-week amphotericin B plus 5-FC arm had the lowest mortality among the treatment arms (Kanyama & Molloy 2017).

*AmBisome plus high dose fluconazole for treatment of HIV-associated cryptococcal meningitis (AMBITION-cm)* - AMBITION-cm is a phase II randomized, controlled, non-inferiority trial that has recently shed some light on the use of a few large doses of liposomal amphotericin B with high dose fluconazole for the treatment of cryptococcal meningitis in HIV patients. The short-course high-dose polyene arms received one, two or three doses of liposomal amphotericin B while being given a high-dose fluconazole backbone regimen. Data has shown the rate of clearance of CSF cultures in all the short-course, high-dose arms to be non-inferior to the standard two-week course of daily polyene, with none of the participants necessitating any treatment interruptions. The single dose (10 mg/kg/day) liposomal amphotericin B regimen is being taken to a phase III trial (Jarvis et al. 2017). These new strategies have the potential to reduce cost and toxicity.

*Pipeline drugs* - APX001 is a first-in-class antifungal compound inhibiting fungal protein Gwt1 with no cross-reaction with the human protein. It has recently been shown that combining APX001 with fluconazole will reduce the fungal burden in mice with cryptococcal meningitis significantly compared to either agent alone. It also has the added advantage of oral bioavailability and even more potent anticryptococcal compounds in this class have been discovered (Schell et al. 2017). AR-12, a celecoxib derivative, repurposed for antifungal therapy, inhibits fungal acetyl-CoA synthase I and down regulates host chaperone proteins that reduce host immune response. It has shown antifungal activity against *Cryptococcus spp.* as well as candida and moulds (Perfect 2017). T-2307, an allylamine that inhibits fungal mitochondrial membrane potential has been shown to have extremely potent antifungal activity *in vitro* and against cryptococcosis in animal models. The azoles have further evolved and with new technology to reduce CYP450 interactions, several compounds, VT1129 and VT1598, have shown outstanding fungicidal activity in murine models of cryptococcosis (Perfect 2017).

In the area of repurposing available drugs, both sertraline and tamoxifen have the potential to be used in combination with existing antifungal drugs for treatment of cryptococcal meningitis and cryptococcosis. In fact, sertraline has been successfully used in a pilot study and a more definitive trial is heading toward completion (Rhein et al. 2016). The calcineurin pathway has also been shown to be an important factor in cryptococcal virulence and utilizing calcineurin inhibitors such as tacrolimus / cyclosporine in combination with antifungal drugs could have potential in the future. However, it is likely that it will be necessary to discover congeners of these landmark drugs that possess more potent antifungal activity but also reduced immunosuppressive activities (Perfect 2017).

*Neurapheresis* - Neurapheresis is a new, potential technology for managing cryptococcal meningitis that is currently in development. Cryptococcal meningitis causes an increase in CSF pressure by mechanical occlusion of the arachnoid villi and thus rapid killing and removing yeasts from the subarachnoid space may change our management of cryptococcal meningitis. Utilizing the size of the encapsulated yeast, neurapheresis involves mechanically filtering the CSF from the subarachnoid space (SAS) using a membrane with pores ≤ 5 μm in diameter to trap the fungus. A peristaltic pump circulates the CSF of rabbits with cryptococcal meningitis through this filter and reintroduces it back into the SAS. After six hours of cycling through the filter, the fungal colony forming units in the CSF were reduced by up to 99%. Further studies may aim at combining neurapheresis with direct antifungal drug delivery such as amphotericin B intrathecally to create high efficacy regimens along with systemic antifungal agents (Cutshaw et al. 2016).

*In conclusion* - Mortality and morbidity still remain high for cryptococcal meningitis despite several strategies for management. Infections in the CNS can be unforgiving and therefore, all tools available must be utilized. Complications such as increased intracranial pressure and IRIS must be recognized and treated despite unclear guidelines. Early diagnosis, expert care (Spec et al. 2017) and rapidly fungicidal regimens will allow more optimal management. However, clearly, the discovery of more potent and less toxic antifungal agents will be helpful to reduce the negative impact of this CNS fungal infection. We are poised today to make an improvement in our antifungal arsenal tomorrow.

## References

[B1] Aberg JA, Price RW, Heeren DM, Bredt B (2002). A pilot study of the discontinuation of antifungal therapy for disseminated cryptococcal disease in patients with acquired immunodeficiency syndrome, following immunologic response to antiretroviral therapy. J Infect Dis.

[B2] Antachopoulos C, Walsh TJ (2012). Immunotherapy of Cryptococcus infections. Clin Microbiol Infect.

[B3] Baddour LM, Perfect JR, Ostrosky-Zeichner L (2005). Successful use of amphotericin B lipid complex in the treatment of cryptococcosis. Clin Infect Dis.

[B4] Beardsley J, Wolbers M, Kibengo FM, Ggayi AB, Kamali A, Cuc NT (2016). Adjunctive dexamethasone in HIV-associated cryptococcal meningitis. N Engl J Med.

[B5] Bicanic T, Bottomley C, Loyse A, Brouwer AE, Muzoora C, Taseera K (2015). Toxicity of amphotericin B deoxycholate-based induction therapy in patients with HIV-associated cryptococcal meningitis. Antimicrob Agents Chemother.

[B6] Bicanic T, Meintjes G, Wood R, Hayes M, Rebe K, Bekker LG (2007). Fungal burden, early fungicidal activity, and outcome in cryptococcal meningitis in antiretroviral-naive or antiretroviral-experienced patients treated with amphotericin B or fluconazole. Clin Infect Dis.

[B7] Bicanic T, Wood R, Meintjes G, Rebe K, Brouwer A, Loyse A (2008). High-dose amphotericin B with flucytosine for the treatment of cryptococcal meningitis in HIV-infected patients: a randomized trial. Clin Infect Dis.

[B8] Boulware DR, Meya DB, Muzoora C, Rolfes MA, Hullsiek KH, Musubire A (2014). Timing of antiretroviral therapy after diagnosis of cryptococcal meningitis. N Engl J Med.

[B9] Brouwer AE, Rajanuwong A, Chierakul W, Griffin GE, Larsen RA, White NJ (2004). Combination antifungal therapies for HIV-associated cryptococcal meningitis: a randomised trial. Lancet.

[B10] Chotmongkol V, Jitpimolmard S (1992). Itraconazole in the treatment of cryptococcal meningitis. J Med Assoc Thai.

[B11] Coker RJ, Viviani M, Gazzard BG, Du Pont B, Pohle HD, Murphy SM (1993). Treatment of cryptococcosis with liposomal amphotericin B (AmBisome) in 23 patients with AIDS. AIDS.

[B12] Collins HL, Bancroft GJ (1992). Cytokine enhancement of complement-dependent phagocytosis by macrophages: synergy of tumor necrosis factor-alpha and granulocyte-macrophage colony-stimulating factor for phagocytosis of Cryptococcus neoformans. Eur J Immunol.

[B13] Cutshaw D, Premji A, Pagadala P, Giamberardino C, McCabe A, Lad SP (2016). A novel therapeutic approach for cryptococcal meningitis. Open Forum Infect Dis.

[B14] Day JN, Chau TTH, Wolbers M, Mai PP, Dung NT, Mai NH (2013). Combination antifungal therapy for cryptococcal meningitis. N Engl J Med.

[B15] Gans J, Portegies P, Tiessens G, Schattenkerk JKE, van Boxtel CJ, van Ketel RJ (1992). Itraconazole compared with amphotericin B plus flucytosine in AIDS patients with cryptococcal meningitis. AIDS.

[B16] Denning DW, Armstrong RW, Lewis BH, Stevens DA (1991). Elevated cerebrospinal fluid pressures in patients with cryptococcal meningitis and acquired immunodeficiency syndrome. Am J Med.

[B17] Desmet P, Kayembe KD, Vroey C (1989). The value of cryptococcal serum antigen screening among HIV-positive/AIDS patients in Kinshasa, Zaire. AIDS.

[B18] Dromer F, Bernede-Bauduin C, Guillemot D, Lortholary O, French Cryptococcosis Study G (2008). Major role for amphotericin B-flucytosine combination in severe cryptococcosis. PLoS ONE.

[B19] Gaskell KM, Rothe C, Gnanadurai R, Goodson P, Jassi C, Heyderman RS (2014). A prospective study of mortality from cryptococcal meningitis following treatment induction with 1200 mg oral fluconazole in Blantyre, Malawi. PLoS ONE.

[B20] George IA, Spec A, Powderly WG, Santos CAQ (2017). Comparative epidemiology and outcomes of HIV, non-HIV non-transplant and organ transplant associated cryptococcosis: a population-based study. Clin Infect Dis.

[B21] Graybill JR, Sobel J, Saag M, van der Horst C, Powderly W, Cloud G (2000). Diagnosis and management of increased intracranial pressure in patients with AIDS and cryptococcal meningitis. The NIAID Mycoses Study Group and AIDS Cooperative Treatment Groups. Clin Infect Dis.

[B22] Hamill RJ, Sobel JD, El-Sadr W, Johnson PC, Graybill JR, Javaly K (2010). Comparison of 2 doses of liposomal amphotericin B and conventional amphotericin B deoxycholate for treatment of AIDS-associated acute cryptococcal meningitis: a randomized, double-blind clinical trial of efficacy and safety. Clin Infect Dis.

[B23] Haubrich RH, Haghighat D, Bozzette SA, Tilles J, McCutchan JA (1994). High-dose fluconazole for treatment of cryptococcal disease in patients with human immunodeficiency virus infection. J Infect Dis.

[B24] (2017). HRSA - Organ Procurement and Transplantation Network.

[B25] Jarvis JN, Leeme TB, Chofle AA, Bidwell G, Molefi M, Tsholo K (2017). Ambition-cm: high-dose liposomal amphotericin for HIV-related cryptococcal meningitis.

[B26] Jarvis JN, Meintjes G, Rebe K, Williams GN, Bicanic T, Williams A (2012). Adjunctive interferon-gamma immunotherapy for the treatment of HIV-associated cryptococcal meningitis: a randomized controlled trial. AIDS.

[B27] Kabanda T, Siedner MJ, Klausner JD, Muzoora C, Boulware DR (2014). Point-of-care diagnosis and prognostication of cryptococcal meningitis with the cryptococcal antigen lateral flow assay on cerebrospinal fluid. Clin Infect Dis.

[B28] Kanyama C, Molloy S (2017). Advancing cryptococcal meningitis treatment for Africa (ACTA).

[B29] Kaplan JE, Vallabhaneni S, Smith RM, Chideya-Chihota S, Chehab J, Park B (2015). Cryptococcal antigen screening and early antifungal treatment to prevent cryptococcal meningitis: a review of the literature. J Acquir Immune Defic Syndr.

[B30] Kawakami K, Qureshi MH, Koguchi Y, Zhang T, Okamura H, Kurimoto M (1999). Role of TNF-alpha in the induction of fungicidal activity of mouse peritoneal exudate cells against Cryptococcus neoformans by IL-12 and IL-18. Cell Immunol.

[B31] Kawakami K, Qureshi MH, Zhang T, Okamura H, Kurimoto M, Saito A (1997). IL-18 protects mice against pulmonary and disseminated infection with Cryptococcus neoformans by inducing IFN-gamma production. J Immunol.

[B32] Kawakami K, Tohyama M, Teruya K, Kudeken N, Xie Q, Saito A (1996a). Contribution of interferon-gamma in protecting mice during pulmonary and disseminated infection with Cryptococcus neoformans. FEMS Immunol Med Microbiol.

[B33] Kawakami K, Tohyama M, Xie Q, Saito A (1996b). IL-12 protects mice against pulmonary and disseminated infection caused by Cryptococcus neoformans. Clin Exp Immunol.

[B34] Larsen RA, Bozzette SA, Jones BE, Haghighat D, Leal MA, Forthal D (1994). Fluconazole combined with flucytosine for treatment of cryptococcal meningitis in patients with AIDS. Clin Infect Dis.

[B35] Larsen RA, Leal MA, Chan LS (1990). Fluconazole compared with amphotericin B plus flucytosine for cryptococcal meningitis in AIDS. A randomized trial. Ann Intern Med.

[B36] Larsen RA, Pappas PG, Perfect J, Aberg JA, Casadevall A, Cloud GA (2005). Phase I evaluation of the safety and pharmacokinetics of murine-derived anticryptococcal antibody 18B7 in subjects with treated cryptococcal meningitis. Antimicrob Agents Chemother.

[B37] Leenders AC, Reiss P, Portegies P, Clezy K, Hop WC, Hoy J (1997). Liposomal amphotericin B (AmBisome) compared with amphotericin B both followed by oral fluconazole in the treatment of AIDS-associated cryptococcal meningitis. AIDS.

[B38] Letang E, Muller MC, Ntamatungiro AJ, Kimera N, Faini D, Furrer H (2015). Cryptococcal antigenemia in immunocompromised human immunodeficiency virus patients in rural Tanzania: a preventable cause of early mortality. Open Forum Infect Dis.

[B39] Liechty CA, Solberg P, Were W, Ekwaru JP, Ransom RL, Weidle PJ (2007). Asymptomatic serum cryptococcal antigenemia and early mortality during antiretroviral therapy in rural Uganda. Trop Med Int Health.

[B40] Longley N, Jarvis JN, Meintjes G, Boulle A, Cross A, Kelly N (2016). Cryptococcal antigen screening in patients initiating ART in South Africa: a prospective cohort study. Clin Infect Dis.

[B41] Martinez E, Garcia-Viejo MA, Marcos MA, Perez-Cuevas JB, Blanco JL, Mallolas J (2000). Discontinuation of secondary prophylaxis for cryptococcal meningitis in HIV-infected patients responding to highly active antiretroviral therapy. AIDS.

[B42] Mayanja-Kizza H, Oishi K, Mitarai S, Yamashita H, Nalongo K, Watanabe K (1998). Combination therapy with fluconazole and flucytosine for cryptococcal meningitis in Ugandan patients with AIDS. Clin Infect Dis.

[B43] McKenney J, Bauman S, Neary B, Detels R, French A, Margolick J (2015). Prevalence, correlates, and outcomes of cryptococcal antigen positivity among patients with AIDS, United States, 1986-2012. Clin Infect Dis.

[B44] Menichetti F, Fiorio M, Tosti A, Gatti G, Pasticci MB, Miletich F (1996). High-dose fluconazole therapy for cryptococcal meningitis in patients with AIDS. Clin Infect Dis.

[B45] Messina JA, Maziarz EK, Spec A, Kontoyiannis DP, Perfect JR (2017). Disseminated cryptococcosis with brain involvement in patients with chronic lymphoid malignancies on ibrutinib. Open Forum Infect Dis.

[B46] Meya DB, Manabe YC, Castelnuovo B, Cook BA, Elbireer AM, Kambugu A (2010). Cost-effectiveness of serum cryptococcal antigen screening to prevent deaths among HIV-infected persons with a CD4+ cell count < or = 100 cells/microL who start HIV therapy in resource-limited settings. Clin Infect Dis.

[B47] Micol R, Lortholary O, Sar B, Laureillard D, Ngeth C, Dousset JP (2007). Prevalence, determinants of positivity, and clinical utility of cryptococcal antigenemia in Cambodian HIV-infected patients. J Acquir Immune Defic Syndr.

[B48] Milefchik E, Leal MA, Haubrich R, Bozzette SA, Tilles JG, Leedom JM (2008). Fluconazole alone or combined with flucytosine for the treatment of AIDS-associated cryptococcal meningitis. Med Mycol.

[B49] Mussini C, Pezzotti P, Miro JM, Martinez E, Quiros JC, Cinque P (2004). Discontinuation of maintenance therapy for cryptococcal meningitis in patients with AIDS treated with highly active antiretroviral therapy: an international observational study. Clin Infect Dis.

[B50] Nelson MR, Bower M, Smith D, Reed C, Shanson D, Gazzard B (1990). The value of serum cryptococcal antigen in the diagnosis of cryptococcal infection in patients infected with the human immunodeficiency virus. J Infect.

[B51] Nussbaum JC, Jackson A, Namarika D, Phulusa J, Kenala J, Kanyemba C (2010). Combination flucytosine and high-dose fluconazole compared with fluconazole monotherapy for the treatment of cryptococcal meningitis: a randomized trial in Malawi. Clin Infect Dis.

[B52] Panackal AA, Komori M, Kosa P, Khan O, Hammoud DA, Rosen LB (2017). Spinal arachnoiditis as a complication of cryptococcal meningoencephalitis in non-HIV previously healthy adults. Clin Infect Dis.

[B53] Panackal AA, Marr KA, Williamson PR (2016). Dexamethasone in cryptococcal meningitis. N Engl J Med.

[B54] Pappas PG, Bustamante B, Ticona E, Hamill RJ, Johnson PC, Reboli A (2004). Recombinant interferon-gamma 1b as adjunctive therapy for AIDS-related acute cryptococcal meningitis. J Infect Dis.

[B55] Pappas PG, Chetchotisakd P, Larsen RA, Manosuthi W, Morris MI, Anekthananon T (2009). A phase II randomized trial of amphotericin B alone or combined with fluconazole in the treatment of HIV-associated cryptococcal meningitis. Clin Infect Dis.

[B56] Perfect JR, Dismukes WE, Dromer F, Goldman DL, Graybill JR, Hamill RJ (2010). Clinical practice guidelines for the management of cryptococcal disease: 2010 update by the infectious diseases society of america. Clin Infect Dis.

[B57] Perfect JR (2016). Editorial commentary: life-saving antimicrobial drugs: what are we doing to pricing and availability?. Clin Infect Dis.

[B58] Perfect JR (2017). The antifungal pipeline: a reality check. Nat Rev Drug Discov.

[B59] Pitisuttithum P, Negroni R, Graybill JR, Bustamante B, Pappas P, Chapman S (2005). Activity of posaconazole in the treatment of central nervous system fungal infections. J Antimicrob Chemother.

[B60] Rajasingham R, Smith RM, Park BJ, Jarvis JN, Govender NP, Chiller TM (2017). Global burden of disease of HIV-associated cryptococcal meningitis: an updated analysis. Lancet Infect Dis.

[B61] Rhein J, Morawski BM, Hullsiek KH, Nabeta HW, Kiggundu R, Tugume L (2016). Efficacy of adjunctive sertraline for the treatment of HIV-associated cryptococcal meningitis: an open-label dose-ranging study. Lancet Infect Dis.

[B62] Robinson PA, Bauer M, Leal MA, Evans SG, Holtom PD, Diamond DA (1999). Early mycological treatment failure in AIDS-associated cryptococcal meningitis. Clin Infect Dis.

[B63] Rolfes MA, Hullsiek KH, Rhein J, Nabeta HW, Taseera K, Schutz C (2014). The effect of therapeutic lumbar punctures on acute mortality from cryptococcal meningitis. Clin Infect Dis.

[B64] Rollot F, Bossi P, Tubiana R, Caumes E, Zeller V, Katlama C (2001). Discontinuation of secondary prophylaxis against cryptococcosis in patients with AIDS receiving highly active antiretroviral therapy. AIDS.

[B65] Saag MS, Powderly WG, Cloud GA, Robinson P, Grieco MH, Sharkey PK (1992). Comparison of amphotericin B with fluconazole in the treatment of acute AIDS-associated cryptococcal meningitis. The NIAID Mycoses Study Group and the AIDS Clinical Trials Group. N Engl J Med.

[B66] Saijo T, Chen J, Chen SC, Rosen LB, Yi J, Sorrell TC (2014). Anti-granulocyte-macrophage colony-stimulating factor autoantibodies are a risk factor for central nervous system infection by Cryptococcus gattii in otherwise immunocompetent patients. MBio.

[B67] Schell W, Giamberardino C, Shaw K, Perfect JR (2017). Efficacy of oral APX001 in a murine model of cryptococcal meningitis.

[B68] Sharkey PK, Graybill JR, Johnson ES, Hausrath SG, Pollard RB, Kolokathis A (1996). Amphotericin B lipid complex compared with amphotericin B in the treatment of cryptococcal meningitis in patients with AIDS. Clin Infect Dis.

[B69] Siddiqui AA, Brouwer AE, Wuthiekanun V, Jaffar S, Shattock R, Irving D (2005). IFN-gamma at the site of infection determines rate of clearance of infection in cryptococcal meningitis. J Immunol.

[B70] Singh N, Lortholary O, Alexander BD, Gupta KL, John GT, Pursell KJ (2005). Antifungal management practices and evolution of infection in organ transplant recipients with Cryptococcus neoformans infection. Transplantation.

[B71] Sloan DJ, Parris V (2014). Cryptococcal meningitis: epidemiology and therapeutic options. Clin Epidemiol.

[B72] Spec A, Olsen MA, Raval K, Powderly WG (2017). Impact of infectious diseases consultation on mortality of cryptococcal infection in patients without HIV. Clin Infect Dis.

[B73] Ssekitoleko R, Kamya MR, Reingold AL (2013). Primary prophylaxis for cryptococcal meningitis and impact on mortality in HIV: a systematic review and meta-analysis. Future Virol.

[B74] Tassie JM, Pepper L, Fogg C, Biraro S, Mayanja B, Andia I (2003). Systematic screening of cryptococcal antigenemia in HIV-positive adults in Uganda. J Acquir Immune Defic Syndr.

[B75] Tenforde MW, Mokomane M, Leeme T, Patel RKK, Lekwape N, Ramodimoosi C (2017). Advanced HIV disease in Botswana following successful antiretroviral therapy rollout: incidence of and temporal trends in cryptococcal meningitis. Clin Infect Dis.

[B76] Thompson GR, Rendon A, Santos RR, Queiroz-Telles F, Ostrosky-Zeichner L, Azie N (2016). Isavuconazole treatment of cryptococcosis and dimorphic mycoses. Clin Infect Dis.

[B77] Vibhagool A, Sungkanuparph S, Mootsikapun P, Chetchotisakd P, Tansuphaswaswadikul S, Bowonwatanuwong C (2003). Discontinuation of secondary prophylaxis for cryptococcal meningitis in human immunodeficiency virus-infected patients treated with highly active antiretroviral therapy: a prospective, multicenter, randomized study. Clin Infect Dis.

[B78] Williamson PR, Jarvis JN, Panackal AA, Fisher MC, Molloy SF, Loyse A (2017). Cryptococcal meningitis: epidemiology, immunology, diagnosis and therapy. Nat Rev Neurol.

[B79] Williamson PR (2017). The relentless march of cryptococcal meningitis. Lancet Infect Dis.

[B80] Yao Y, Zhang JT, Yan B, Gao T, Xing XW, Tian CL (2015). Voriconazole: a novel treatment option for cryptococcal meningitis. Infect Dis.

[B81] Zhou Q, Gault RA, Kozel TR, Murphy WJ (2006). Immunomodulation with CD40 stimulation and interleukin-2 protects mice from disseminated cryptococcosis. Infect Immun.

[B82] Zolopa A, Andersen J, Powderly W, Sanchez A, Sanne I, Suckow C (2009). Early antiretroviral therapy reduces AIDS progression/death in individuals with acute opportunistic infections: a multicenter randomized strategy trial. PLoS ONE.

